# Exercise, headache, and factors associated with headache in chronic whiplash: Analysis of a randomized clinical trial

**DOI:** 10.1097/MD.0000000000018130

**Published:** 2019-11-27

**Authors:** Maria Landén Ludvigsson, Gunnel Peterson, Simon Widh, Anneli Peolsson

**Affiliations:** a Department of Medical and Health Sciences, Division of Physiotherapy; b Rehab Väst, Östergötland County Council, Department of Rehabilitation and Department of Medical and Health Sciences, Linköping University; c Centre for Clinical Research Sörmland, Uppsala University; d Rörelse Hälsa, County Council of Östergötland, Department of Medical and Health Sciences, Linköping University, Linköping, Sweden.

**Keywords:** chronic, exercise, headache, rehabilitation, whiplash

## Abstract

**Background:**

Almost 40% of individuals with chronic whiplash-associated disorders (WAD) report headache after 5 years, making it one of the most common persistent symptoms besides neck pain, but randomized treatment studies are lacking. This study aimed to evaluate the effect of 3 different exercise approaches on headache in chronic WAD grades 2 and 3, and to identify potential factors associated with such headache, and whether they differ depending on 3 different aspects of such headache (current headache, maximum headache, or headache bothersomeness).

**Methods:**

This was an analysis of a randomized clinical trial of people with chronic WAD and headache (n = 188), who were randomized to either 12 weeks of neck-specific exercise without (NSE) or with a behavioral approach (NSEB) or physical activity prescription (PPA). Data were collected at baseline and at 3, 6, and 12 months. Physical and psychosocial factors were tested for association with headache. Multivariate regression models and linear mixed models were used.

**Results:**

The NSE/NSEB groups reported reduced headache both over time and compared to PPA. Up to 51% (NSE) and 61% (NSEB) reported at least 50% reduction in their headache at 12 months. The PPA group was not improved over time. Neck pain and dizziness were associated with headache regardless of aspect of headache. The only associated psychosocial factor was anxiety, which was associated with headache bothersomeness. Other factors were mainly physical, and up to 51% of the variance was explained.

**Conclusion:**

Headache in chronic WAD, may be reduced with neck-specific exercise with or without a behavioral approach. Chronic headache was associated with neck pain and dizziness regardless of aspect tested. Other factors associated with headache in chronic WAD were mainly physical rather than psychosocial.

**Trial registration number:**

Clinical Trials.gov, no: NCT015285

## 1 Introduction

Headache is one of the most common causes of years lived with disability, with a prevalence of more than 10% of the global population.^[1]^ One cause of persistent headache (>3 months) can be a whiplash injury,^[2]^ which in itself presents a significant public health problem with an incidence of at least 300 per 100,000.^[3]^ A whiplash injury can follow a sudden incidence that causes acceleration-deceleration forces to act on the neck. Multiple anatomical sites in the neck have been postulated for a whiplash injury, including for instance neck muscles, facet joints, spinal ligaments, and intervertebral discs.^[4]^ Remaining symptoms after a whiplash injury (whiplash-associated disorders [WAD]) are reported in up to 50% of the cases after 1 year,^[5]^ and after 5 years, nearly 40% continue to report headache, making it one of the most common problems in addition to neck pain in WAD.^[6]^ Headache in chronic WAD can be classified as either “persistent headache attributed to whiplash” or “cervicogenic headache” (caused by a disorder of the cervical spine).^[2]^ Headache in subacute WAD has been associated with several features, for example, neck pain, temporomandibular disorders,^[7]^ neck disability, psychological distress such as depression and anxiety,^[8]^ brain structural abnormalities, and overuse of headache medications.^[2]^ However, to our knowledge factors associated with headache in chronic WAD grade 2 and 3 (2 = pain and local neck findings, 3 = as grade 2 plus neurological signs)^[9]^ have not been explored.

Management of headache associated with neck pain, in general, should include exercise.^[10]^ Improvements in cervicogenic headache have been reported following both strength and endurance training including neck-specific exercise (NSE).^[11,12]^ However, what kind of exercise would be optimal in chronic WAD grade 2 and 3 remains unclear. It is well known that characteristic morphological changes and altered muscle behaviour in cervical muscles are specific features of WAD.^[13–18]^ Ligaments reportedly account for only 25% of the cervical stability^[19]^ and the deep muscles thus have an important task in maintaining the vertebrae within a neutral position where loading is optimally distributed over all supporting structures.^[20]^ NSE may thus have an important positive impact on cervicogenic headache in WAD. However, also psychosocial factors have been attributed to the persistence of symptoms in individuals with WAD.^[21]^ Furthermore, combinations of cervicogenic headache and other types of headaches are common.^[22]^ Patients who for instance suffer from chronic tension-type headaches may also benefit from relaxation training.^[10]^

In chronic WAD grades 2 and 3, NSE has reportedly positive effects of on several outcomes including neck pain and disability,^[23]^ neck muscle endurance,^[24]^ and cost-effectiveness.^[25]^ To our knowledge, there are however no randomized controlled trials which have reported the effect of different exercise interventions on headache in chronic WAD. The aim of this secondary analysis was 2-fold: to evaluate the effect of 3 different exercise approaches on headache in chronic WAD grades 2 and 3, and to evaluate potential factors associated with headache in chronic WAD, and whether they differ depending on 3 different aspects of headache (headache bothersomeness, current, or maximum headache).

## 2 Methods

### 2.1 Designs and procedure

This is a secondary analysis of a multicentre randomized clinical trial of exercise in chronic WAD grade 2 and 3.^[26]^ Informed consent was collected before randomization, which was carried out using a computer-generated list. An independent researcher put the results into opaque envelopes for further distribution to the treating physiotherapists. The data, collected with questionnaires and physical tests at baseline and at 3, 6, and 12 months, was registered by another independent blinded researcher, and all tests were performed by researchers (experienced physiotherapists) blinded to allocation. The procedures were conducted according to the Declaration of Helsinki, and the study was approved by the Regional Ethics Committee of Linköping University, Sweden.

### 2.2 Interventions and setting

All treating physiotherapists worked within primary care in 6 Swedish counties, and were selected to match their interests and knowledge as far as possible. They participated in a 1-day workshop focusing on the content of the specific intervention in question and were provided with standardized oral and written study information.

The 3 interventions were undertaken during a 12-week period. The time frame and specific components of the interventions have been previously published,^[23,27]^ but are presented briefly below.

#### 2.2.1 NSE

NSE with a focus on the deep cervical muscles, was performed with a physiotherapist twice weekly, with additional home exercises. The first step was unresisted activation of the deep muscles. When this was performed correctly without activation of the superficial muscles, gym exercises without pain provocation were introduced, with progressive head resistance training in a weighted pulley, focusing on good posture and low load endurance. A detailed description of the exercises can be found in the Academic Archive On-line.^[28]^

#### 2.2.2 Neck-specific exercise with a behavioral approach (NSEB)

The exercises were the same as those undertaken by the NSE group, but in accordance with the concept of graded exercise, participants were encouraged to focus on success in exercise progression, and not temporary increase in local neck pain.^[29]^ Behavioral interventions including 2 pain education sessions (1 repetition of key messages), and introductions to activities aimed at pain management (eg, relaxation, breathing exercises) and problem solving were also provided.

#### 2.2.3 Prescription of physical activity (PPA)

Based on a short motivational interview and a physical examination, participants were prescribed individualized general physical activity (eg, gym classes, Nordic walking), to be performed outside the health care system.^[30]^ No NSEs of the deep muscles were prescribed. One follow-up visit or phone call was encouraged.

### 2.3 Participants

**Figure 1:**
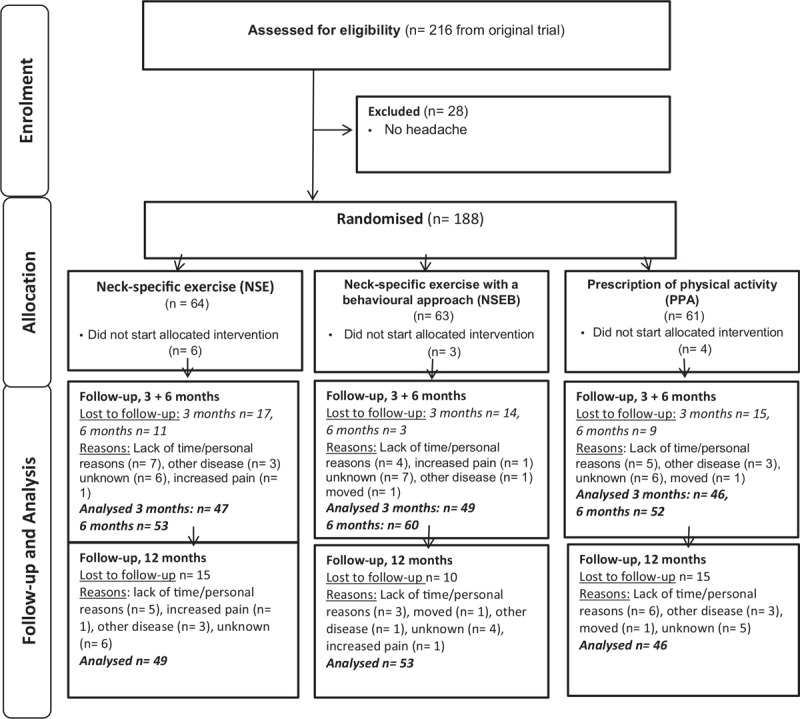
Flow chart of participants.

Participants who reported headache in the preceding week of >2 mm on a visual analog scale (VAS) scale, and headache at least from time to time on a 5-grade scale from never to constantly (as previously used in studies of neck surgery),^[31]^ were included in this analysis (n = 188 out of 216 in the main study, Fig. 1). Pain <3 mm on a 100 mm VAS scale has been defined as no pain.^[32]^ There were 128 females and 60 males, with a mean age of 40 (SD12) years (Table 1). Participants were recruited in 2011 to 2012, and follow-ups were completed in 2013. Further inclusion criteria included: WAD grade 2 or 3 for 6 to 36 months, a neck disability index (NDI)^[33]^ score of >10/50 points, and/or an average neck pain intensity over the past week on the VAS of >20/100 mm. Exclusion criteria included: more dominant pain elsewhere, previous neck trauma with unresolved symptoms, fractures or other conditions that were potentially detrimental to completing the study interventions or insufficient knowledge of the Swedish language.^[26]^

**Table 1:**
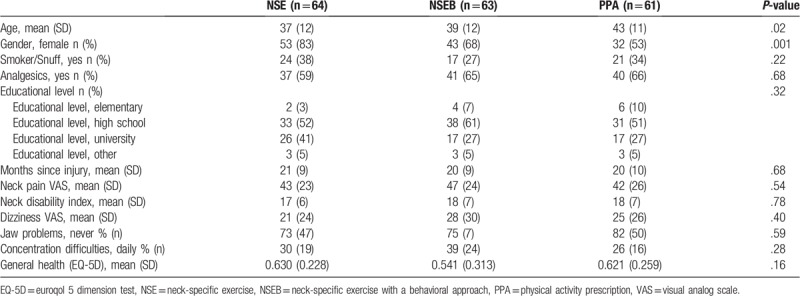
Baseline characteristics of study participants.

### 2.4 Outcome measurements

The primary outcome was headache as reported on 3 different VAS scales (0–100 mm): current headache, maximum headache in the preceding week, and headache bothersomeness in the preceding 24 hours. Exercise diaries were also collected to monitor adherence.

Baseline factors considered as potential factors associated with headache in chronic WAD were selected based on a review of the literature and on the clinical experience^[34]^ of 3 specialist physiotherapists with over 20 years’ experience of managing patients with neck pain including WAD. The chosen factors were: age, gender, neck pain, dizziness (VAS 0–100), jaw problems and difficulties concentrating (from never to constantly on a 5-grade scale), educational level, smoking, physical activity level (International Physical Activity Questionnaire),^[35]^ general health euroqol 5 dimension test,^[36]^ pain catastrophizing (Pain Catastrophizing Scale),^[37]^ depression and anxiety (Hospital Anxiety and Depression Scale),^[38]^ balance between effort and reward at work (Effort-Reward Imbalance),^[39]^ self-efficacy of performing various activities despite pain (Self-Efficacy Scale),^[40]^ neck flexor and extensor muscle endurance,^[41]^ total active sagittal motion and difference between sides in lateral flexion and rotation in degrees, measured with a Cervical Range-Of-Motion device.^[42]^

### 2.5 Statistics

The sample-size calculation was based on the primary outcome, NDI, in the main study (3.5/50, SD7, alpha 5%, power 80%).

Descriptive statistics were calculated, and between-group comparisons at baseline were evaluated with the Kruskal–Wallis test for nonparametric data, with the Mann–Whitney *U* test for post-hoc, or with 1-way analysis of variance for normally distributed parametric data. In dichotomous outcomes, Chi-square tests were used.

The main analyses were carried out on an intention-to-treat basis, including all available data at each time point. Linear mixed models (LMM) were used to analyze the 3 normally distributed VAS change-scores from baseline to 3 months, baseline to 6 months, baseline to 12 months (time), and group (3 levels; NSE, NSEB, PPA). A compound symmetry heterogenous covariance matrix was used. Overall time (all groups together), group (general mean difference between intervention groups), and a group-by-time interaction were included as fixed factors and age as covariate. LMM was favored since all the data available at all time points can be included. However, unlike the change scores, the baseline scores were not normally distributed and did not fulfil the assumptions of LMM. The within group median changes from baseline to 12 months were thus performed with Kruskal–Wallis’ tests with Mann–Whitney *U*-test for post-hoc. In addition to these analyses, the proportion of participants with >50% reduction in pain (=treatment success or substantial improvement), in this case headache, is presented as recommended by the initiative on methods, measurement, and pain assessment in clinical trials.^[43]^

Spearman or Pearson correlation tests, as appropriate, were used for correlations with baseline headache and the potential associated factors. The significant factors were checked one-by-one for multicollinearity with the linear regression technique before being included in the final regression model. Residuals for the dependent variables (VAS scales) were normally distributed. A variance inflation factor (VIF) >10 or tolerance level <0.1 were used to denote significant multicollinearity,^[44]^ and no collinearity was denoted (all VIF <3.2, tolerance >0.33). Multivariable regression models using stepwise backward regression with *P* ≥ .1 as a limit for removal of variables to reduce the risk of overlooking potentially relevant factor combinations were performed.

The significance level was set at *P* ≤ .05. Analyses were performed with SPSS 23.0.

## 3 Results

Loss to follow-up was 24% (3 months), 12% (6 months), and 21% (12 months) (Fig. 1). There was no difference between drop-outs and follow-ups regarding any of the pain outcomes or gender but drop-outs were somewhat younger (36 vs 40 years old at 12 months, *P* = .04).

**Figure 2:**
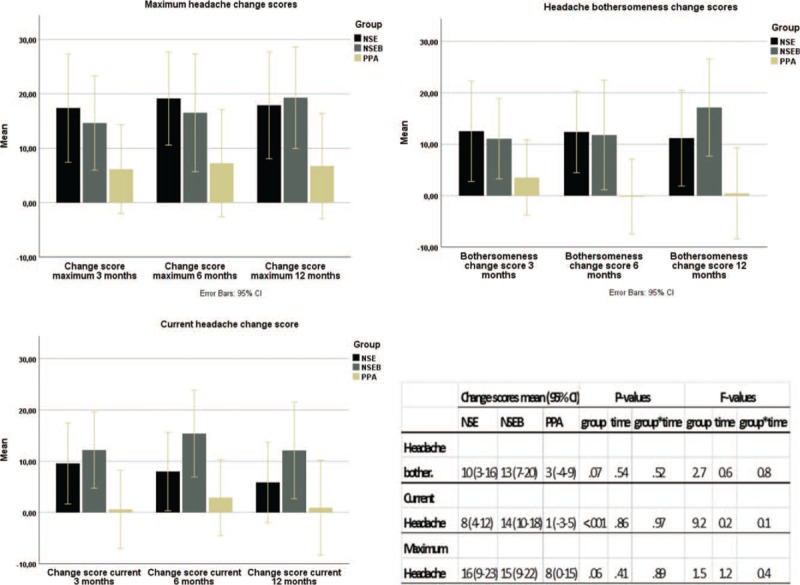
Change scores from baseline to 3, 6, and 12 mo. Results of the linear mixed models. NSE = neck-specific exercise, NSEB = neck-specific exercise with a behavioral approach, PPA = prescription of physical activity.

There was a significant mean group difference in change scores between groups in current headache (*P* < .001, Fig. 2) while the other 2 outcomes did not quite reach significance (*P* = .06, and .07). A post-hoc difference was reported favoring the NSEB group over the PPA group (*P* < .01), but the difference between the NSE and PPA group did not quite reach significance (*P* = .06). Regarding time, there was no significant difference in change scores from 3 to 12 months. All group-by-time interactions were insignificant (Fig. 2). However, when comparing within-group median changes from baseline, the NSEB group was improved over time at all time points and VAS measurements and the NSE group was improved in headache bothersomeness and maximum headache. The PPA group reported no improvement over time (Table 2). There were no differences between the NSE/NSEB groups in any of the outcomes.

**Table 2:**
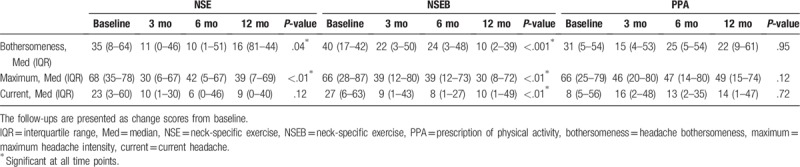
Median baseline scores and improvement over time in 3 different measures of headache.

**Figure 3:**
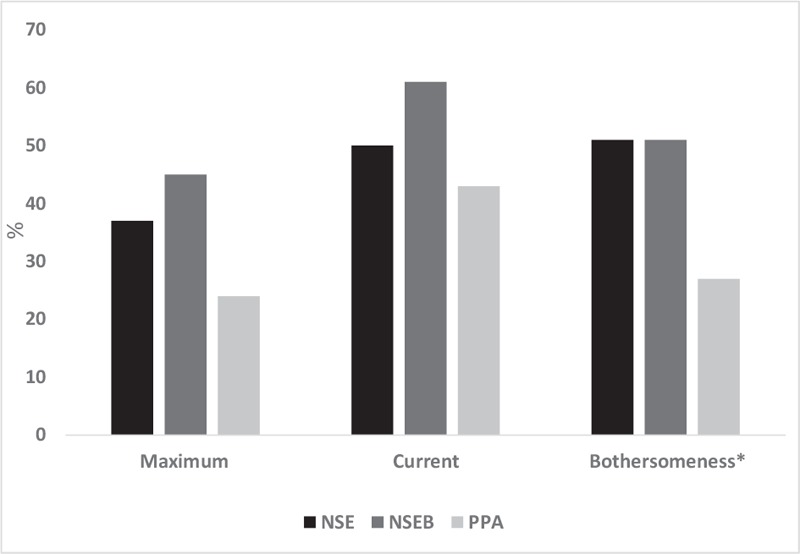
Percentage of participants with at least 50% reduction of headache at 12 mo. ^∗^*P* < .05. NSE = neck-specific exercise, NSEB = neck-specific exercise with a behavioral approach, PPA = prescription of physical activity maximum = maximum headache, current = current headache, bothersomeness = headache bothersomeness.

There was also a significantly higher proportion of participants who reported at least 50% reduction in headache bothersomeness in the NSE/NSEB groups versus PPA at 12 months (50% vs 27%, *P* < 0.05). There was no significant difference between groups regarding the proportion of participants with 50% reduction in current or maximum pain (Fig. 3).

No serious harm was registered but 2 participants dropped out due to increased pain (Fig. 1).

There was a moderate to strong correlation between the 3 different measures of headache (Table 3) and several of the tested factors were correlated with headache in the bivariate correlation test preceding the multivariate regression model. Taking all these significant factors into account in the multivariate regression models, higher neck pain and more dizziness were the only factors associated with all 3 headache outcomes, whereas for instance higher anxiety was only associated with bothersomeness. None of the other psychosocial factors were part of the explained variance which tended to be mainly physical (Table 4). In the final models, 43% to 51% of the variance regarding headache was explained.

**Table 3:**
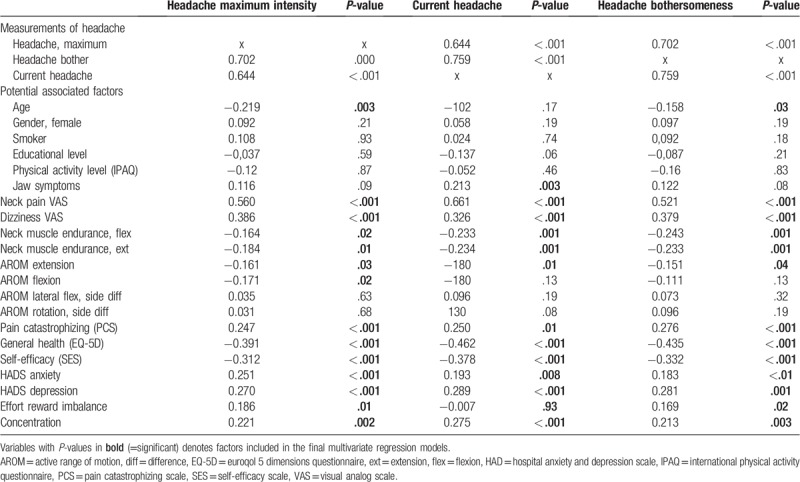
Bivariate correlation of headache measurements, and potential factors associated with headache.

**Table 4:**
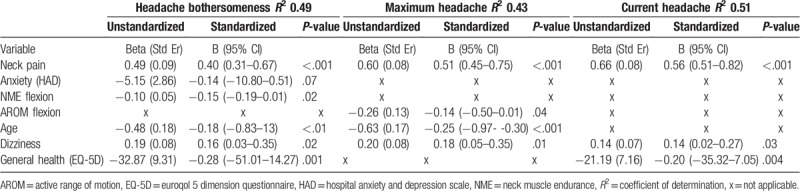
Results of the final multivariate regression model of factors associated with headache.

## 4 Discussion

The results of this analysis indicate that for people with chronic WAD (in this sample with a mean duration of around 2 years) NSE, or NSEB may reduce headache compared to PPA. The results seem to be lasting, as underpinned by the insignificant mean time difference between the change scores at 3, 6, and 12 months. Change scores are calculated as the mean change from baseline to each time point. This change score, but not the actual baseline values, are included in the LMM analysis, which is why the insignificant time factor in the LMM thus indicates that the results remained unchanged over time between the first to the last follow-up. As analyzed in the Kruskal–Wallis’ test, the NSE/NSEB groups were improved in time from baseline in the median VAS scores whereas the PPA group reported no significant improvements. There was a significant mean group difference in change scores between groups in current headache (*P* < .001, Fig. 2), where the NSEB group reported higher change scores than the PPA-group. The other 2 outcomes did not quite reach a significant difference between groups but there was a strong trend for the higher change scores for NSE/NSEB groups (*P* = .06, and .07). In the NSE/NSEB groups, 51% of the participants (Fig. 3) reported at least a 50% reduction in their headache bothersomeness after 12 months, defining substantial improvement or treatment success.^[43]^ This was significantly higher than in the PPA-group (27%). Pain reduction is one of the most important outcomes for people with chronic pain,^[45]^ and taking into account the fact that little change can be expected to occur spontaneously after 3 months’ duration in chronic WAD,^[9,46]^ the results of this study are promising. The clinical relevance of improvements in low ratings at baseline can be discussed. However, the mean reduction in pain among those who reported at least 50% improvement was quite high: 45 mm (SD 26) in maximum headache, 28 mm (SD 25) in current headache, and 35 mm (SD 25) in headache bothersomeness.

Since the exercise was neck-specific and there was no difference between the NSE/NSEB groups, this could indicate that the headache in many cases was cervicogenic without substantial psychosocial influence. This was also underpinned by the results of the multivariate analysis of factors associated with headache, where the only psychosocial factor that remained in the final step was anxiety. It was then only associated with bothersomeness as one of the factors explaining the variance, but not with current or maximum pain. Clinical tests were conducted both as part of the test protocol, and by the treating physiotherapists, confirming neck pain and other common symptoms in chronic WAD. There were; however, no tests specifying the type of headache in this study. Nonetheless, the results of this study are consistent with the positive results previously reported in general cervicogenic headache, following NSE.^[11,12]^

In the bivariate correlation analyses, there were both physical and psychosocial factors that correlated with the headache outcomes, and they were similar regardless of outcome (bothersomeness, current pain, or maximum headache). In the final multivariate regression models, when all significant factors were included and controlled for, higher levels of neck pain and dizziness were the 2 important factors that partly explained the variance of all 3 outcomes. Lower active range of neck motion or neck muscle endurance in flexion, lower age, and lower general health were also part of the explained variance in 2 out of 3 outcomes. The 3 different aspects of pain had a high correlation, and similar factors also correlated with these 3 different aspects of pain, suggesting that either of these VAS measurements may be used to evaluate headache in chronic WAD. If compared to reported factors in the acute stage associated with developing chronic headache, the only factors reported to be the same as the ones found in chronic WAD in this study were anxiety^[8]^ and neck pain.^[47]^ Partly this could be due to some factors tested were not the same in those studies. Predictors in the acute phase, like a sore throat and a lack of confidence in recovering completely, were not included in our study, whereas other factors like neck muscle endurance, general health, several psychosocial measurements, and more were included.

There were no significant improvements for the PPA group. However, it should be noted that the PPA group also had a rather high number of participants who reported more than 50% reduction in their headache at 12 months, in particular in current headache.

Headache was present in 87% of the participants in the original study, from which the participants were included. This is more than presented in the general chronic WAD population.^[6]^ One possible explanation is that this study did not include WAD grade 1 (neck pain only without clinical findings), but only the more severe grades 2 and 3.^[9]^ Even though jaw problems correlated with current headache in the bivariate tests, this was not part of the explained variance. This may be because 79% reported no jaw problems at all. Dizziness was, however, reported in 74% and the association between dizziness and headache was confirmed in the multivariate regression models. This indicates that people with headache in chronic WAD should also be screened for dizziness, which may also be improved with NSE.^[48]^ It was surprising that gender was not associated with headache in WAD like in many other types of headache. Therefore, a comparison between genders was also performed, which confirmed that there was no significant difference between genders (all *P* > .16).

There were more women than men in this study, which is consistent with the general WAD population.^[49]^ An analysis of individuals that fulfilled the criteria but declined to participate in this study showed that the study sample was well representative in terms of gender, age, and level of neck pain as previously presented.^[23]^ Results from a multicentre study, like this one, may also be more generalizable to physiotherapy practice in a range of primary-care settings than a single center study.

### 4.1 Limitations

There are some limitations to this study that should be recognized. This is a secondary analysis of a randomized trial and the sample size was not based on headache. Still, some of the results demonstrated significantly better results for the NSE/NSEB groups which indicates enough power for these results. There were also some results that were close to significant, where a few more participants probably would have been needed to demonstrate a significant difference.

For maximum headache in the previous week, there is a risk of recall bias, which could have an impact on the level of pain. However, since the question was the same at all time points, it most likely did not influence the results.

There was a baseline difference between groups regarding age and gender, but gender was not correlated with headache in any of the outcomes, and age was controlled for in the analyses. Analgesics were not included as a variable, since the question used in the questionnaire was directed at neck pain, not headache. However, the use of analgesics was reduced in the neck-specific group as previously reported.^[23]^

Finally, even though up to 51% of the variance was explained in the multivariate regression models, other factors, not tested in this study, may also be associated with headache in chronic WAD grade 2 and 3. It is, however, a strength of this study that variables from both clinical tests and questionnaires were included in this analysis.

## 5 Conclusion

Headache in chronic WAD grade 2 and 3, maybe reduced with neck-specific exercise with or without a behavioral approach. Chronic headache was associated with neck pain and dizziness regardless of aspect tested. Other factors associated with headache in chronic WAD were mainly physical rather than psychosocial.

## Acknowledgments

The authors thank all participants in this study, including WAD participants, physiotherapists, and staff involved at any stage of the study.

## Author contributions

**Conceptualization:** Gunnel Peterson, Anneli Peolsson.

**Data curation:** Maria Landén Ludvigsson, Gunnel Peterson.

**Formal analysis:** Maria Landén Ludvigsson, Simon Widh.

**Funding acquisition:** Anneli Peolsson, Gunnel peterson and Maria Landén Ludvigsson.

**Methodology:** Maria Landén Ludvigsson, Gunnel Peterson, Anneli Peolsson.

**Project administration:** Maria Landén Ludvigsson, Gunnel Peterson, Anneli Peolsson.

**Supervision:** Maria Landén Ludvigsson, Anneli Peolsson.

**Writing – original draft:** Maria Landén Ludvigsson.

**Writing – review and editing:** Gunnel Peterson, Simon Widh, Anneli Peolsson.

Maria Landén Ludvigsson orcid: 0000-0002-3259-3133.

Anneli Peolsson's Orcid Id 0000-0002-6075-4432.

Gunnel peterson's Oric 0000-0003-2492-0306.
